# Effect of Compressive Stress in Tumor Microenvironment on Malignant Tumor Spheroid Invasion Process

**DOI:** 10.3390/ijms23137091

**Published:** 2022-06-25

**Authors:** Ryota Nishi, Yudai Oda, Takashi Morikura, Shogo Miyata

**Affiliations:** 1 Graduate School of Science and Technology, Keio University, Yokohama 223-8522, Japan; ryota.ff.hb.2408@gmail.com (R.N.); dnngu-1elife@keio.jp (T.M.); 2 Department of Mechanical Engineering, Faculty of Science and Technology, Keio University, Yokohama 223-8522, Japan; yudai_oda@keio.jp

**Keywords:** mechanobiology, tumor, spheroid, colorectal cancer, invasion

## Abstract

In this study, we proposed an in vitro tumor model to simulate the mechanical microenvironment and investigate the effect of compressive stress on the invasion process of malignant tumors. It has been pointed out that the biomechanical environment, as well as the biochemical environment, could affect the transformation of cancer cell migration, invasion, and metastasis. We hypothesized that the solid stress caused by the exclusion of surrounding tissue could transform tumor cells from noninvasive to invasive phenotypes. Colorectal cell spheroids were embedded and cultured in agarose gels of varying concentrations to simulate the earliest stages of tumor formation and invasion. The spheroids embedded in gels at higher concentrations showed peculiar growth after 72 h of culture, and the external compressive loading imposed on them caused peculiar growth even in the gels at lower concentrations. In conclusion, the mechanical microenvironment caused the transformation of tumor cell phenotypes, promoting the growth and invasion of tumor cell spheroids.

## 1. Introduction

Colorectal cancer substantially reduces the 5-year survival rate of patients and is the 4th most commonly diagnosed malignancy and the 3rd most common cause of cancer deaths worldwide, with approximately 2 million new cases and almost 1 million deaths in 2018. Its burden is expected to increase by 60% by 2030 [1]. The frequency of metastasis in colorectal cancer is high, making it extremely important to diagnose and treat the disease at an early stage before malignant tumor cells begin to invade the surrounding tissues.

It is widely known that the biochemical environment is closely related to the transformation of cancer cell migration and invasion. Recently, it has been shown that the interaction between tumors and the surrounding mechanical microenvironment influences the migration transformation and invasiveness of cancer cells [2,3]. In colorectal cancer, the gastrointestinal tract itself can be deformed by physiological activities, subjecting colorectal cancer cells to various endogenous mechanical stresses. Furthermore, during the progression of cancer, the rapid proliferation of tumor cells combined with the inhibition of apoptosis by cell carcinogenesis leads to a rapid increase in compressive stress acting on malignant tumor tissue [4]. Simultaneously, deposition of the extracellular matrix (ECM) and collagen cross-linking around the tumor tissue increases its rigidity [5]. As described above, extremely complex mechanical microenvironments can be constructed around the colorectal cancer, where the mechanical microenvironment affects the colorectal cancer cells at all stages of cancer growth, including their development, migration, transformation, invasion, and metastasis.

Several in vitro and in situ studies have reported that the mechanical microenvironment surrounding cancer tissue affects its invasion and progression. Previous studies on colorectal cancers have reported that the mechanical stiffness of cells adapts to changes in the mechanical microenvironment to alter the proliferation and invasiveness of cells [6]. Taubenberger et al. also reported that confining breast cancer cell spheroids using polyethylene glycol (PEG) hydrogels of different stiffness altered cell proliferation and invasiveness by adapting their mechanical stiffness based on the rigidity of the surrounding tissue [7,8]. However, these studies focused on the metastatic stage of cancer cells with vigorous proliferation and invasiveness. Moreover, it remains unclear what role the mechanical microenvironment plays during the earliest stages of cancer cell development, from the formation of cell aggregates to tumor growth and the transformation from a non-invasive cell phenotype to an invasive phenotype.

We hypothesized that during the earliest stages of colorectal cancer development, when the solid stress from the surrounding tissue due to the growth of cancer cell spheroids exceeded a certain critical limit, the migration phenotypes would be transformed and invade the surrounding tissue. In this study, we constructed a confined three-dimensional tumor model in which colorectal cancer spheroids were embedded in agarose gels of different stiffnesses. Agarose gel does not contain cell adhesion molecules to suppress the interaction with the surrounding matrix because the tumor cells in the earliest stages exhibit strong cell–cell interactions and are isolated from surrounding tissues [4]. Using this confined tumor model, we examined the effect of solid stress caused by the exclusion of the surrounding tissues on spheroid growth. By varying the mass concentration of the agarose gel, the solid stress from the surrounding matrix could be changed, and the effect of it on the growth and gene expression of tumor cells could be evaluated. Furthermore, an external compressive load was imposed on the spheroids via hydrogels to confirm the relationship between the mechanical environment and invasiveness of colorectal cancer spheroids during the earliest stages of colorectal cancer development.

## 2. Materials and Methods

### 2.1. Fabrication of Tumor Spheroids Confined in Agarose Gels

Human colorectal cancer cells (DLD-1, Riken Bioresource Center, Ibaraki, Japan) were used to construct cell spheroids. The DLD-1 cell spheroids were embedded in agarose gel to simulate a tumor confined in the surrounding tissue in vivo. To simulate the earliest stages of tumor development and invasion, the tumor spheroids were embedded in agarose gels of different mass concentrations—that is, 0.5, 0.75, 1.0, 1.5, and 2.0%. The DLD-1 cells were cultured in RPMI medium supplemented with 10% fetal bovine serum (FBS), 1% antimycotic-antibiotic solution under 5% CO_2_, 37 °C, and 95% humidity conditions. The cells were passaged 2–3 times before spheroid fabrication. To fabricate the cell spheroids, expanded cells were collected using 0.25% trypsin and seeded in agarose gel microwells made using a commercial rubber template (3D Petri Dish, Sigma-Aldrich, MO, USA) at a concentration of 4.0 × 10^5^ cells/mL. After 15 min of incubation in a CO_2_ incubator to sediment the cells in each microwell, the cells were cultured with 1 mL of fresh culture medium for 1 d to form spheroids.

**Figure 1:**
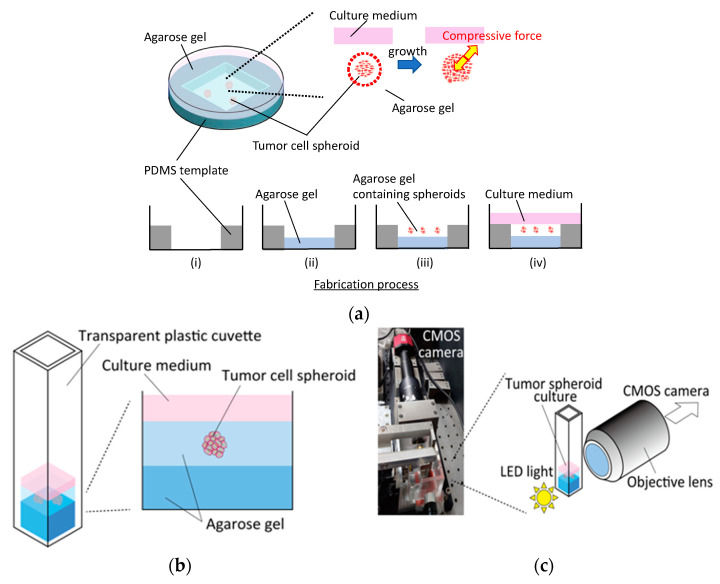
Fabrication and observation procedures of tumor spheroid culture confined in agarose gels. Fabrication process of (**a**) a petri-dish-based culture and (**b**) a transparent cuvette-based culture with (**c**) a custom microscopy system to enable observation from the side of the cuvette.

To enable the phase-contrast and fluorescent microscopy processes, and to observe the spheroid growth during external compression, we proposed two types of confined tumor culture models using a Petri dish and a disposable transparent cuvette. To construct the Petri-dish-based culture model, a polydimethylsiloxane (PDMS) template containing a 30 mm^2^ hole was set in a *ϕ*60-mm dish and 600 mL of agarose gel solution at each concentration was poured and gelled at 4 °C for 15 min to prevent cell adhesion to the dish (Figure 1a). Before encapsulation in the agarose gel, the spheroids were collected from the agarose gel microwells and resuspended in the agarose solution at each concentration. After gelation of the basal agarose gel layer, 400 mL of agarose solution containing spheroids was poured to overlay the basal layer. The gel layer containing spheroids was then incubated at 4 °C for 15 min to embed and confine the spheroids in the agarose gel. In this study, 0.5, 0.75, 1.0, 1.5, and 2.0% agarose gels were used to vary the stiffness of the gels. Finally, 1.5 mL of fresh culture medium was filled to overlay the gel layer containing the spheroids. To prepare control specimens, spheroids under free-swelling conditions were cultured in the agarose gel microwells. Phase-contrast microscopy images were obtained at 0, 24, 48, and 72 h after spheroid embedding in the agarose gel. Fluorescence microscopy images were obtained after 72 h of culture. The growth and viability of the tumor spheroids in agarose gels at different concentrations were evaluated using the microscopy images.

A transparent, disposable cuvette (Fisher Scientific, Hampton, NH, USA) was used to construct the cuvette-based culture model. As was the case with the dish-based cultures, 300 µL of the agarose gel solution at each concentration was poured and gelled at 4 °C for 15 min to fabricate the base gel (Figure 1b). After gelation, 150 µL of the agarose solution containing spheroids was poured onto the basal gel and incubated at 4 °C for 15 min. The spheroids in the cuvette were then cultured in 210 µL fresh medium. To enable observation from the side of the cuvette, a custom-made microscopy system was developed by assembling a 10× objective lens (Olympus, Tokyo, Japan), LED light, and CMOS camera (CS2100M-USB, Thorlabs, Newton, NJ, USA) (Figure 1c). Bright-field images were obtained at 0 and 72 h after gel fabrication. The effect of the externally applied compressive stress on the growth direction of spheroids was examined using the microscopy images.

### 2.2. Imposing External Compressive Stress on Tumor Spheroids by Mechanical Loading of Agarose Gel

**Figure 2:**
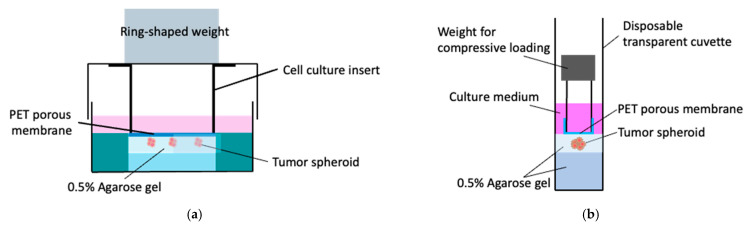
Tumor spheroid culture subjected to external compressive stress using transparent PET membrane and ring-shaped weight. (**a**) Petri-dish-based culture and (**b**) cuvette-based culture.

To confirm the relationship between the peculiar spheroid growth and the solid stress generated by the gel exclusion due to spheroid growth, the external compressive stress via the compression of the agarose gel was imposed on the tumor spheroids confined in the agarose gel at a low concentration (0.5%). The agarose gel was subjected to compressive loading via a microporous membrane with a weight based on our previous reports [9,10]. For the petri dish culture, the agarose gel was subjected to compressive loading using an 8 μm pore size cell culture insert (BD Falcon Inc., East Rutherford, NJ, USA) with a ring-shaped weight to enable oxygen and nutrient exchange and observation of the spheroids through the microporous membrane (Figure 2a). To compensate for the difference in solid stress between 0.5 and 1.5% agarose gels, an SUS304 stainless steel ring-shaped weight was placed on the lid of the dish to apply an additive compressive stress of 5.5 × 10^2^ Pa, which is the difference in solid stress between 0.5 and 1.5% agarose gels, to the spheroids in the 0.5% agarose gel. Similarly, for the cuvette-based culture, a polycarbonate pipe (inner diameter: 5 mm, outer diameter: 8 mm, length: 10 mm) covered with an 8 mm pore size polyester (PET) membrane was fabricated to simulate the microporous membrane-based culture insert (Figure 2b). To impose a compressive stress of 5.5 × 10^2^ Pa, a SUS304 stainless steel ring-shaped weight was placed on the polycarbonate pipe. The external compressive stress, 5.5 × 10^2^ Pa, was determined based on the numerical analysis described in Section 2.4.

### 2.3. Morphometric Analysis of Tumor Spheroids

**Figure 3:**
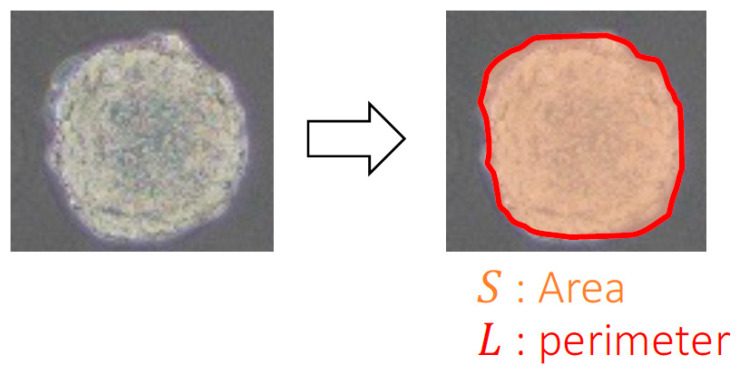
Quantitative analyses for spheroid growth using phase-contrast microscopy images.

To evaluate the peculiar growth and invasiveness of spheroids, the area and circularity of the spheroids in the phase-contrast microscopy images were examined. Briefly, the outlines of the spheroids were manually extracted, and the areas and circularity were analyzed using image analysis software (ImageJ, NIH, Bethesda, MD, USA). To evaluate the growth of the spheroids, the increase in the ratio of the area compared to the area at 0 h was calculated as follows:

(1) $$increase\;ratio\;of\;speroid\;area=\frac{S}{S_{0}}$$

where *S*_0_ is the total area of the spheroids at 0 h and *S* is the area measured at each time point (Figure 3). The circularity *K* of the spheroids was calculated as follows:

(2) $$K=\frac{4\pi \times S}{L^{2}}$$

where *L* is the perimeter at each time point (Figure 3).

### 2.4. Numerical Analysis of Growth-Induced Compressive Force on the Spheroids

**Figure 4:**
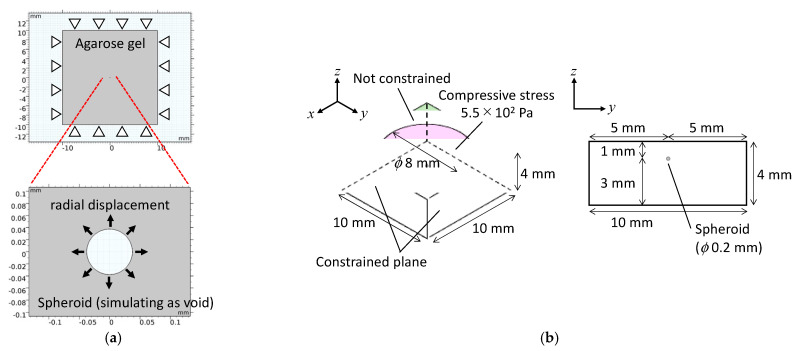
Numerical analysis models for (**a**) the petri-dish-based and (**b**) the cuvette-based spheroid culture models with external compressive stress.

Numerical analyses were conducted using finite element software (COMSOL Multiphysics Version 5.2a, COMSOL Inc., Stockholm, Sweden). To determine the solid compressive stress on the tumor spheroids by exclusion of the surrounding agarose gel, a two-dimensional numerical model was constructed to simulate a spheroid embedded in agarose gel. In the model, a perfect circular void simulating the spheroid was positioned in a square matrix (Figure 4a). The solid stress generated by the exclusion of the agarose gel was calculated by applying radial displacement to the surface of the voids to simulate the isotropic growth of cultured spheroids. The diameter of the void was set to 188 µm, which was the mean value of the measured diameter from the phase-contrast images at 0 h. The geometry of the square matrix simulating the agarose gel was 20 × 20 mm. The material parameters of the agarose gels of varying concentrations are listed in Table 1. The elastic modulus of each agarose gel was determined using a custom-made material testing device (Figure S1). The radial displacements applied to the void surface were set based on the measured diameters from the phase-contrast images on day 2.

A three-dimensional numerical model was constructed to analyze the mechanical environment of the spheroids subjected to external compressive loading. In the three-dimensional model, the agarose gel was modeled as a rectangular body (10 × 10 × 4 mm^3^), and the spheroid was modeled as a sphere (diameter: 200 µm) positioned at the centroid of the rectangular body (Figure 4b). Material parameters of the agarose gel were defined based on the experimental data (Table 1) and previous studies—that is, Young’s modulus, Poisson’s ratio, and the mass concentration of the 0.5% agarose gel were 2.7 kPa, 0.49 [11], and 1.0 × 10^3^ kg/m^3^, respectively. Young’s modulus, Poisson’s ratio, and the mass concentration of the spheroid were also defined as 4.2 kPa [12], 0.36 [13], and 1.0 × 10^3^ kg/m^3^, respectively. A compressive stress of 5.5 × 10^2^ Pa was imposed on the circular area on the top surface of the rectangular body to simulate the experimental conditions. The side and lower surfaces of the rectangular body were constrained.

### 2.5. RT-PCR Analysis

The expression of mRNA, *COL1A2* encoding collagen type I alpha 2, was quantified by reverse transcription quantitative polymerase chain reaction (RT-qPCR) in the spheroid and monolayer cell cultures. It has been reported that the *COL1A2* gene was expressed markedly in the mesenchymal phenotype of the DLD-1 cells to increase migration and invasive activity [14]. The total RNA of specimens cultured for 72 h was extracted using a NucleoSpin RNA (740955.50; Takara Bio Inc., Shiga, Japan) and quantified using a Thermal Cycler Dice Real-Time System Lite (TP700; Takara Bio Inc.). The RNA was reverse transcribed into cDNA using a PrimeScript Master Mix (Perfect Real Time) (RR036A; Takara Bio Inc.) with an oligo (dT) primer and random hexamer primer for 15 min at 37 °C and 5 s at 85 °C. The concentration of cDNA was quantified using a biophotometer (6131; Eppendorf, Hamburg, Germany) and then diluted using RNase-free water (9012; Takara Bio Inc.) to 10 ng/µL of cDNA. RT-qPCR was conducted in a Thermal Cycler Dice Real-Time System Lite for 30 s at 95 °C and 60 cycles of 5s at 95 °C and 30s at 60 °C. The RT-qPCR mixed reagent contained 12.5 µL of TB Green Premix Ex Taq II (Tli RnaseH Plus) (RR820A; Takara Bio Inc.), 20 ng of cDNA, 0.4 µM of each forward and reverse primer, and 8.5 µL of RNase-free water. The primer sequences used are listed in Table 2. RT-qPCR was performed in triplicate for each primer pair and cDNA sample. Moreover, the reactions were conducted in triplicate under similar conditions. To verify that the primer dimers were not responsible for the obtained fluorescence signals, melting curve analysis of the amplicons was conducted. Negative control reactions without templates were performed to ensure data quality. The relative mRNA expression was normalized to *GAPDH* before being calibrated using the quantity relative to the quantity obtained from the spheroids under free-swelling conditions. The fold-change was calculated using the 2^−ΔΔC^_T_ method, where C_T_ is the threshold cycle.

### 2.6. Statistical Analysis

Most data are representative of three individual experiments using different cryopreserved cell stocks. For each experimental group, 4–10 samples (*n* = 4–10) were analyzed, with each data point representing the mean and standard deviation. The statistical significance of the experimental data was evaluated using the Tukey–Kramer test or Student’s *t*-test. The statistical significance was set at *p* < 0.05.

## 3. Results

### 3.1. Effect of Hydrogel Stiffness on Invasion Process of Tumor Spheroid

**Figure 5:**
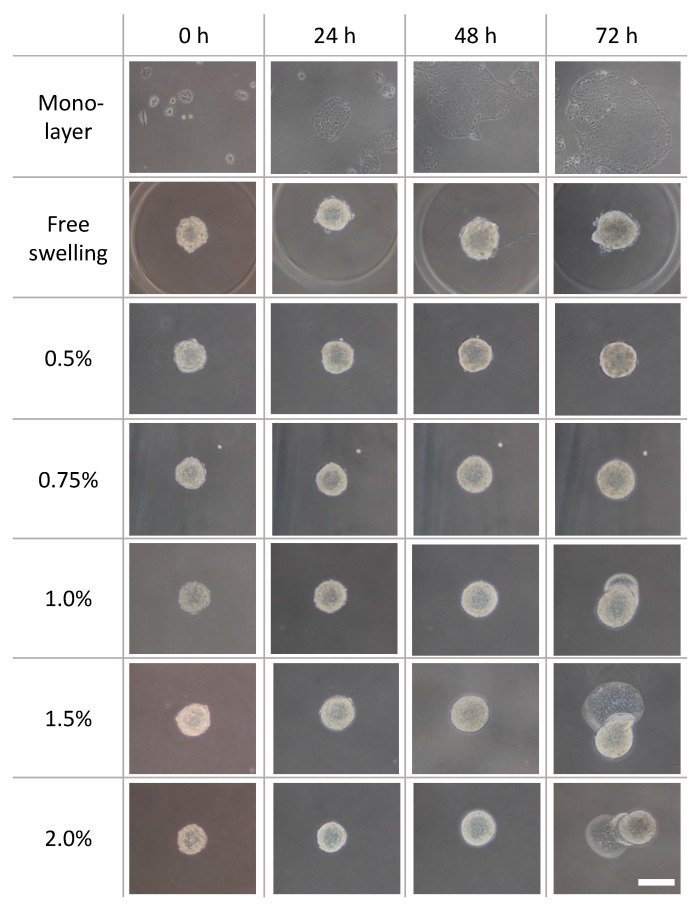
Phase-contrast images of the DLD-1 cell spheroids cultured in agarose gels with different mass concentrations. Scale bar: 200 µm.

**Figure 6:**
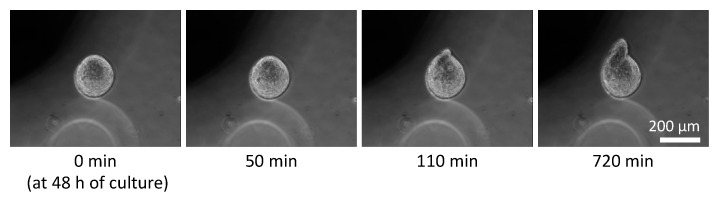
Time course of peculiar growth of tumor spheroids cultured in the 2.0% agarose gel. Scale bar: 200 µm.

**Figure 7:**
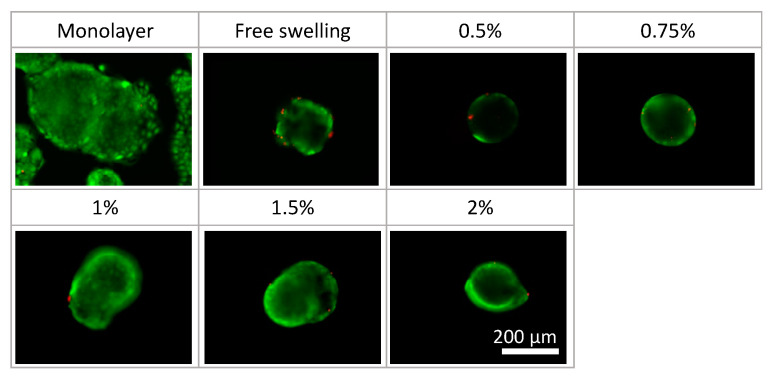
Cell viability in the tumor spheroid culture under different conditions. Scale bar: 200 µm.

Phase-contrast microscopy images of the monolayer culture, spheroids under free-swelling conditions, and spheroids confined in agarose gels are shown in Figure 5. Under all culture conditions, the area occupied by the spheroids increased with the increasing culture period. In the confined spheroid culture, the spheroids in the 0.5% and 0.75% agarose gels showed a concentric and isotropic increase in the spheroid area over the entire culture period. The spheroids in the 1.0%, 1.5%, and 2.0% agarose gels showed a similar concentric increase in spheroid area as the spheroids inside the 0.5% and 0.75% agarose gels until 48 h of culture. Subsequently, peculiar growth of the spheroids was observed as a protrusion forming on the surface of the spheroids after 72 h of culture. The protruding part of the spheroids were formed within a short period of time (approximately 1 h), before cell aggregations were formed around the protruding part (Figure 6, Video S1). Furthermore, the viability of the DLD-1 cells in the spheroids was maintained in all experimental groups after 72 h of culture (Figure 7). This result indicates that the stiffness of the surrounding matrix did not affect cell viability in the confined three-dimensional tumor culture model.

**Figure 8:**
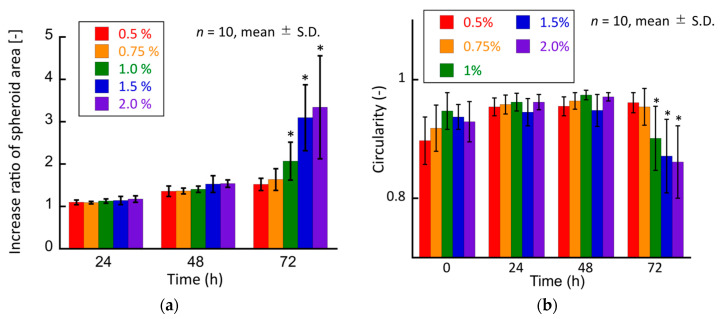
Quantitative analyses of the tumor spheroids cultured in agarose gels. (**a**) Increase ratio of the spheroid area and (**b**) circularity of the spheroid. * indicates a significant difference compared to the 0.5% agarose gel groups (*p* < 0.05).

Moreover, to quantitatively evaluate the growth of cancer cell spheroids in the hydrogel, the increase ratio of the spheroid area was evaluated. This ratio in the 1.0%, 1.5%, and 2.0% agarose gels was considerably higher than that in the 0.5% agarose gel (Figure 8a). The circularity of the spheroids in all experimental groups did not change until day 2 (Figure 8b). Considering the increase in the area of the cultured spheroids, it could be considered that the spheroids grew isotropically under all experimental conditions until 48 h of culture. Conversely, the spheroids in the 1.0%, 1.5%, and 2.0% agarose gels showed a substantial decrease in circularity after 72 h of culture. Considering the increase in the projection area of the spheroids, the spheroids in these experimental groups grew in a non-isotropic and specific manner from 48 to 72 h.

### 3.2. Relationships between Spheroid Growth and Growth-Induced Compressive Force from Surrounding Hydrogel

**Figure 9:**
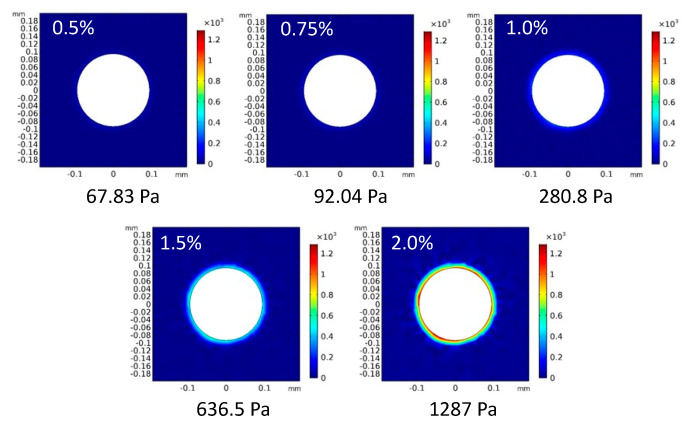
Solid stress distribution and maximum values of von Mises stress in the numerical analysis model.

The stress field around the tumor spheroids was determined by numerical analysis assuming that the diameter of spheroids increased based on the isotropic growth of spheroids until 48 h of culture. The diameter of the spheroids was measured by extracting outlines from the phase-contrast microscopy images, and the increase ratio of the diameter at 48 h to that at 0 h was evaluated. The increase ratios of the spheroids in the 0.5, 0.75, 1.0, 1.5, and 2.0% agarose gels were 116, 117, 118, 122, and 124%, respectively. Based on the spheroid growth, the surrounding agarose gels were isotropically deformed and pushed away from the initial boundary between the gels and spheroids. The spheroids were subjected to solid stress from the surrounding gels toward their centers. The solid stress determined by numerical analysis increased with the increasing concentration of agarose (Figure 9). The values of solid stress were 67.83, 92.04, 280.8, and 1287 Pa for the 0.5, 0.75, 1.0, 1.5, and 2.0% agarose gels, respectively.

### 3.3. Effect of External Compressive Force on Growth of Spheroids

**Figure 10:**
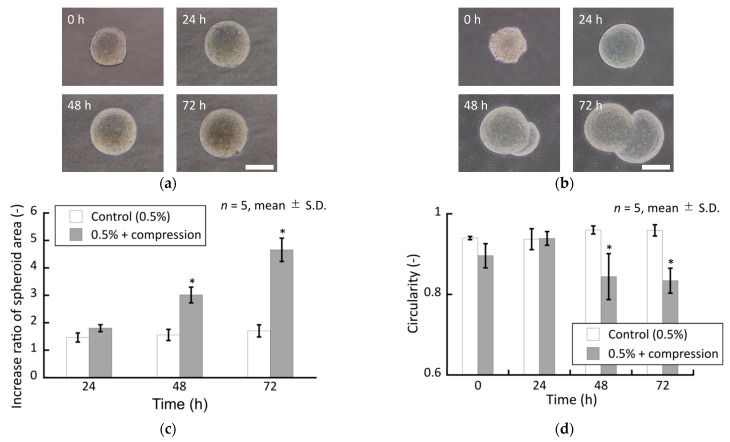
Phase-contrast images of the DLD-1 cell spheroids cultured in the 0.5% agarose gel (**a**) without and (**b**) with external compressive loading. Scale bar: 200 µm. Quantitative characterization of the cultured spheroids. (**c**) Increase ratio of spheroid area and (**d**) circularity of spheroids. * indicates a significant difference compared to control groups (*p* < 0.05).

To verify the effect of solid stress generated by increasing spheroid size on the spheroid growth in the stiffer agarose gels, an external compressive stress was applied to spheroids confined in the 0.5% agarose gel. The spheroids in the 0.5% agarose gel subjected to superimposed compressive stress exhibited peculiar growth from 48 h to 72 h, similar to that observed in the 1.5% and 2.0% agarose gels without superimposed compressive stress (Figure 10).

**Figure 11:**
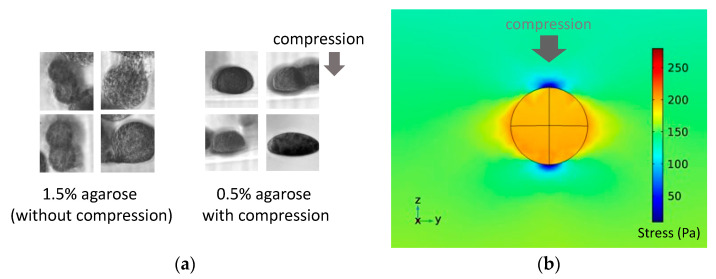
(**a**) Photomicrograph of tumor spheroids in the 0.5% agarose gel observed from the vertical side of the cuvette and (**b**) distribution of the von Mises stress from the numerical analysis simulating the external compressive loading on a spheroid in the agarose gel.

Furthermore, observations from the vertical side of the compressive direction showed that the spheroids in the 1.5% agarose gel without external compression grew in a random direction, whereas those in the 0.5% agarose gel subjected to external compression grew along the horizontal direction (Figure 11a). From the results of the numerical analysis, the stress generated by the external compression of the gel was at its maximum at the outermost horizontal edge of the spheroid (Figure 11b).

### 3.4. Attenuation of Gene Expression of Tumor Spheroids in Hydrogels

**Figure 12:**
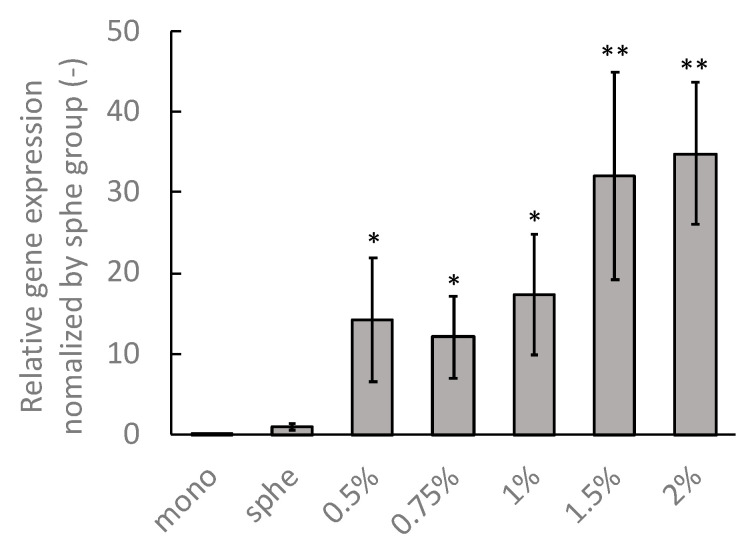
mRNA expression of the DLD-1 cells in the monolayer (mono) spheroids cultured under free-swelling conditions (sphe), and spheroids cultured in agarose gel of different mass concentrations (0.5–2%). * indicates a significant difference compared to the sphe group (*p* < 0.05). ** indicates a significant difference compared to the 0.5% group (*p* < 0.05).

To evaluate the phenotypic transition of the DLD-1 cells in the spheroids confined in the agarose gels, the *COL1A2* gene—which was expressed markedly at the mesenchymal phenotype—was evaluated [14]. The expression of the *COL1A2* gene in the cells under monolayer culture was lower than that in the spheroids under all culture conditions (Figure 12). Furthermore, the gene expression in the spheroids confined in the agarose gel was significantly higher than that under free-swelling conditions. The gene expression in the spheroids confined in the agarose gels tended to increase with increasing agarose concentrations, plateauing at concentrations beyond 1.0% agarose gel.

## 4. Discussion

The aim of this study was to establish an in vitro model that simulates the earliest stages of cancer development and its transition to the invasive phase. Specifically, we investigated the relationship between the mechanical microenvironment and the transformation of non-invasive cell phenotypes to invasive phenotypes in tumor spheroids during the earliest stages of colorectal cancer. Previous studies have reported the external and internal mechanical environments of cancer cell spheroids [7,8,15]. However, their reports used hydrogels containing cell-adhesive matrixes to confine spheroids. Consequently, these culture models focused on the invasive phase of cancer and did not simulate the earliest stages of its development, when the tumor spheroids exhibited strong cell–cell interactions and were isolated from the surrounding matrixes. Helmlinger et al. reported the effect of solid stress on the growth of tumor spheroids [16]. They used differentiated cancer cells to establish an in vitro tumor model to simulate the invasive stages of cancer. In our culture model, the DLD-1 cell line, which could be used to simulate the epithelial-mesenchymal transition process [14], was used to generate tumor spheroids during the earliest stages of cancer development. Furthermore, the spheroids were confined in agarose gels containing no cell-adhesive matrixes to simulate isolation from the surrounding ECMs of cancer during the earliest stages.

Our experimental results showed that the growth of tumor spheroids increased as the stiffness of the surrounding agarose gel increased. Specifically, the tumor spheroids exhibited peculiar growth when the agarose gel concentration around the spheroids exceeded 1.0%. Our results did not agree with those of a previous study which reported that increasing the stiffness of the surrounding gels inhibited the growth of spheroids [16]. It could be speculated that this disagreement in the effect of the gel stiffness on the spheroid growth was due to the difference in cancer cells expressing different phenotypes. In our study, the DLD-1 cells showed an epithelial-like phenotype and could undergo phenotypic transition under solid stress. From the gene expression analysis, the *COL1A2* gene expression in the DLD-1 cell spheroids confined in agarose gels was larger than that in the monolayer culture and spheroids under free-swelling conditions. Furthermore, gene expression increased with increasing agarose concentration. It has been reported that the *COL1A2* gene has been expressed in DLD-1 cells that acquire the invasive and migratory phenotype [14]. Consequently, it could be considered that the DLD-1 cells—subjected to solid stress with spheroid growth—acquired mesenchymal phenotypes that increased their invasiveness and migratory ability.

To confirm the role of solid stress in the progression of tumor spheroids, an external compressive stress was imposed on the 0.5% agarose gel containing the DLD-1 cell spheroids to reach a total solid stress similar to that of the 1.5% agarose gel. From observations using phase-contrast microscopy, the tumor spheroids subjected to external compressive stress in the 0.5% agarose gel exhibited peculiar growth similar to that in agarose gels of higher concentrations. Consequently, solid compressive stress could be considered to be one of the triggers causing the phenotypic transition of tumor cells. Many studies have reported the relationship between the metastatic/invasive potential of cancer cells and the mechanical environment [17,18,19]. Furthermore, in vivo compressive stress induces tumorigenesis [20]. However, these studies did not focus on phenotypic transitions to increase invasive and migratory potential during the earliest stages of tumorigenesis. Our results suggest that the mechanical environment caused a phenotypic transition, resulting in the peculiar growth of the tumor spheroids.

## 5. Conclusions

In conclusion, an in vitro confined tumor model was established to culture tumor spheroids in agarose gels of varying mass concentrations. The spheroids embedded in the agarose gels at all mass concentrations grew isotropically until 48 h, whereas the spheroids in the gels at higher concentrations exhibited peculiar growth. From the results of the numerical analyses, it could be surmised that the solid stress generated by the exclusion of the surrounding gel progressed the peculiar growth of the spheroids. The expression of the *COL1A2* gene—which was observed in mesenchymal phenotypic cells—increased with increasing agarose gel stiffness. Furthermore, external compressive loading induced the peculiar growth of spheroids embedded in the agarose gel at lower concentrations. Moreover, the additional compressive stress induced a peculiar growth similar to that observed in spheroids embedded in agarose gels at higher concentrations and affected the direction of spheroid growth. Finally, it could be suggested that solid compressive stress triggered and progressed tumor growth with phenotypic transition.

## Figures and Tables

**Table 1:** Material parameters of the agarose gels with different mass concentrations.

Agarose	Young’s Modulus (kPa)	Poison Ratio (–)	Mass Concentration (kg/m^3^)
0.5	2.71	0.5	1.0 × 10^3^
0.75	3.58		
1.0	9.93		
1.5	18.2		
2.0	34.5		

**Table 2:** RT-qPCR primer sequences used for gene expression analysis.

Gene Name	Gene Bank Number	Sequence(5′–3′)
*Col1a2*	NM_000089.4	Forward GAGGGCAACAGCAGGTTCACTTA
		Reverse GCACCGTCAAGGCTGAGAAC
*Gapdh*	NM_002046.7	Forward TCAGCACCACCGATGTCCA
		Reverse TGGTGAAGACGCCAGTGGA

## Data Availability

The data that support the findings of this study are available from the corresponding author upon reasonable request.

## Supplementary Materials

The following supporting information can be downloaded at: https://www.mdpi.com/article/10.3390/ijms23137091/s1.
