# Supplementary material for: Effect of Compressive Stress in Tumor Microenvironment on Malignant Tumor Spheroid Invasion Process
Source: Int J Mol Sci. 2022 Jun 25;23(13):7091. doi: 10.3390/ijms23137091 (PMC9266885; doi:10.3390/ijms23137091)
Supplement: Supplementary file 1 [file ijms-23-07091-s001.zip › IJMS-Figure-S1.pdf]

International Journal of
Molecular Sciences

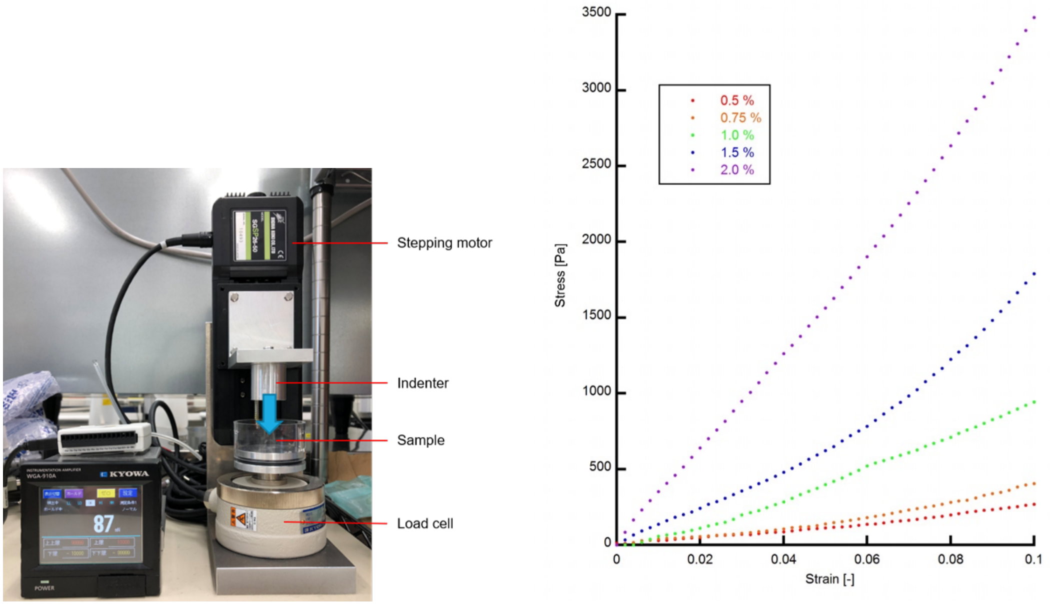

(**a**) (**b**)

Figure S1 (**a**) Experimental set-up for compression test of the agarose gels and (**b**) stress-strain curve for agarose gels of different mass concentrations.
